# NAD(P)H-Hydrate Dehydratase- A Metabolic Repair Enzyme and Its Role in *Bacillus subtilis* Stress Adaptation

**DOI:** 10.1371/journal.pone.0112590

**Published:** 2014-11-13

**Authors:** Miroslava Petrovova, Jan Tkadlec, Lukas Dvoracek, Eliska Streitova, Irena Licha

**Affiliations:** 1 Department of Genetics and Microbiology, Faculty of Science, Charles University, Prague, Czech Republic; 2 Department of Medical Microbiology, 2nd Faculty of Medicine, Charles University, Prague, Czech Republic; Laurentian University, Canada

## Abstract

**Background:**

One of the strategies for survival stress conditions in bacteria is a regulatory adaptive system called general stress response (GSR), which is dependent on the SigB transcription factor in *Bacillus* sp. The GSR is one of the largest regulon in *Bacillus* sp., including about 100 genes; however, most of the genes that show changes in expression during various stresses have not yet been characterized or assigned a biochemical function for the encoded proteins. Previously, we characterized the *Bacillus subtilis*168 osmosensitive mutant, defective in the *yxkO* gene (encoding a putative ribokinase), which was recently assigned *in vitro* as an ADP/ATP-dependent NAD(P)H-hydrate dehydratase and was demonstrated to belong to the SigB operon.

**Methods and Results:**

We show the impact of YxkO on the activity of SigB-dependent P*ctc* promoter and adaptation to osmotic and ethanol stress and potassium limitation respectively. Using a 2DE approach, we compare the proteomes of WT and mutant strains grown under conditions of osmotic and ethanol stress. Both stresses led to changes in the protein level of enzymes that are involved in motility (flagellin), citrate cycle (isocitrate dehydrogenase, malate dehydrogenase), glycolysis (phosphoglycerate kinase), and decomposition of Amadori products (fructosamine-6-phosphate deglycase). Glutamine synthetase revealed a different pattern after osmotic stress. The patterns of enzymes for branched amino acid metabolism and cell wall synthesis (L-alanine dehydrogenase, aspartate-semialdehyde dehydrogenase, ketol-acid reductoisomerase) were altered after ethanol stress.

**Conclusion:**

We performed the first characterization of a *Bacillus subtilis*168 knock-out mutant in the *yxkO* gene that encodes a metabolite repair enzyme. We show that such enzymes could play a significant role in the survival of stressed cells.

## Introduction

In an effort to understand the global adaptation network that evolved in *Bacillus* sp., several recent studies were carried out, focused on the genome-wide transcriptional profiling of the stress response of *Bacillus subtilis* 168 [1]–[4]. Several physiological analyses of the *Bacillus subtilis* 168 proteome during the adaptation to various environmental stresses have been published as well [5]–[7]. These studies identified stress specific regulons that are involved in stress function and confirm that the synthesis of most vegetative proteins is repressed, with the exception of enzymes that take part in adaptive responses.

One of the important strategies for survival in the genus *Bacillus* is a regulatory adaptive system called general stress response (GSR). It occurs as the large expression of stress proteins and is induced by a wide range of stresses, including high and low temperature; osmotic, ethanol, oxidative, and acidic stress; the addition of some antibiotics; starvation for glucose, phosphate, and oxygen; and blue or red light [2], [8]–[12] It is also induced on the transition into the stationary phase [13] and provides cells unspecified, multiple, and preventive resistance and gives the cells sufficient time for the induction of specific stress responses.

The general stress regulon, dependent on the SigB factor, is one of the largest operons in *Bacillus* sp., including about 100 genes [4]. However, most of the genes that show changes in expression during various stresses have not yet been characterized or assigned a biochemical function for the encoded proteins, and the evidence of the contribution of individual proteins from the general stress regulon to stress resistance of *Bacillus subilis* 168 cells is not complete.

Many genes of this regulon are putative regulatory factors, and all are under complex regulation by the control of other sigma factors and other regulatory proteins or RNAs, which allows their complex networking. It is assumed that their role is to protect DNA, proteins, metabolites, and lipids against the harmful effects of stress and to repair them.

Most recently, it was shown by Young [14] that the extent of stress determines response specificity and that the general stress response pathway activates different genes to a variety of stress conditions.

With the aim of elucidating the mechanism of adaptation of *Bacillus subtilis* to limited concentrations of potassium, we previously isolated a mutant with reduced salt tolerance only at a limited potassium concentration [15] in which the *yxkO* gene was interrupted. The product of this gene was formerly predicted to have a ribokinase activity based on sequence and structural homologies and the presence of ATP- and Mg^2+^-binding sites [16]. Most recently, while experiments of this work were completed, the biochemical activity of the YxkO protein was assigned *in* *vitro* as an ADP/ATP-dependent NAD(P)H-hydrate dehydratase (EC 4.2.1.93). This enzyme convert abnormal metabolite NAD(P)H hydrate (NAD(P)HX) to NAD(P)H and is conserved over the kingdoms [17]. NAD(P)HX is slowly catalyzed from NAD(P)H by glyceraldehyde 3-phosphate dehydrogenase [18] or is produced non enzymatically in the course of the non-physiological conditions respectively [19], [20]. NAD(P)HX is unable to react as cofactor and it inhibits several dehydrogenases with detrimental effect on a cell [20], [21]. Enzymes with such activity are called metabolite repair or metabolite-proofreading enzymes and play a role similar to the proofreading activities of DNA polymerases and aminoacyl-tRNA synthetases [22].

The increased transcriptional activity of this gene after osmotic, heat, and ethanol stress was observed in the transcriptomic study of Petersohn [3], as well as in a recent extensive systematic and quantitative exploration of transcriptome changes in *Bacillus subtilis* [4]. The mutant in this gene was included in a phenotype screening study determining the contribution of individual SigB-dependent genes of unknown function to stress resistance, showing a lower survival rate following severe ethanol, heat, and osmotic stresses [23]. Most recently, it was shown to be under exclusive SigB regulation [24].

In accordance with our prior study and in addition to the reduced tolerance to the environmental stresses mentioned above, the mutant in the *yxkO* gene exhibits reduced growth under potassium limitation and altered motility under hyperosmotic conditions. This multiple effect of the gene disruption on phenotype led us originally to the hypothesis that the product of the *yxkO* gene has a regulatory function.

The present study aimed to determine the contribution of the *yxkO* gene product to stress adaptation by estimating the transcriptional activity of the P*ctc* promoter, as the Ctc protein is considered a marker of general stress response [11], and by discovering the changes of the cytoplasmic protein level pattern in the mutant and a wild-type strain when exposed to salt and ethanol stress.

## Materials and Methods

### Bacterial strains

The *Bacillus subtilis* strains and plasmids used in this study are listed in Table 1. *Escherichia coli* DH5α strain (*deoR endA1 gyrA96 hsd R17* (*r_k_* ^−^, *m_k_* ^−^) *recA1 relA1 supE44 thi-1* (*lacZYA-arg F*) *U169 φ80lacZ M15* F^−^λ^−^) (Clontech) was used for propagation of plasmid constructs.

**Table 1:** *Bacillus subtilis* strains, phage, and plasmids used in this study.

Name or code	Relevant genotype or description	Reference, Source, or construction
**Strains**		
B.s. 168 (WT)	*trpC2*	BGSC (1A1)
B.s. SG4 (WT)	*xglA1*, *xglR1*	BGSC (1A680)
L-42	*phe*A1, *spo*0F221, *trp*C2, mini-Tn*10*::*yxkO*	[15]
LD1	*trp*C2, mini-Tn*10*::*yxkO*	this study
MP2	Mutin4::*yxk*O, *trpC*, *xglA1*, *xglR1*	this study
WT/P*ctc*	(P*ctc*Φ *spoVG*-*lacZ*, *cat)*::*amy*, *trpC*, *xglA1*, *xglR1*	this study
MP2/P*ctc*	Mutin4::*yxkO*, (P*ctc*Φ spoVG-*lacZ*, *cat*)::*amy*, *trpC*, *xglA1*,*xglR1*	this study
**Phage** PBS1	Bacillusphage PBS1	BGSC (1P1)
**Plasmids**		
pDG1661	*spoVG-lacZ*, *spc*, *cat*, *bla*, *amy* 5′ and 3′ segment	BGSC (ECE112)
pMUTIN	*spoVG-lacZ*, *Pspac*, *lacI*, *erm*, *bla*	BGSC (ECE139)
pJT2	pDG1661 with *Pctc* insert transcriptionally fused to*spoVG-lacZ*	this study
pMP2	pMUTIN with 5′ end segment of *yxkO* with SD site fusedto *spoVG-lacZ*	this study

### Growth conditions

For genetic manipulations, *Escherichia coli* and *Bacillus subtilis* strains were cultivated routinely in LB medium.

For growth rate measurements, the *Bacillus subtilis* strains (WT, LD1 and MP2) were cultivated under vigorous agitation at 37°C and synchronized in exponential growth by reinoculation from overnight cultures (grown in LB medium) to LB medium for ethanol stress experiments or to MM medium with 0.5 mM K^+^ (as described previously [15]) for both ethanol and osmotic stress experiments, respectively. The salt stress conditions were performed by the exposure of exponentially growing cells (OD_600_ of ∼0.3) to 0.6 M NaCl. For the ethanol stress setup, ethanol to a final concentration of 4% (v/v) was added at the same growth condition as for the salt stress. For the MP2 strain, erythromycin (0.3 µg/ml) was added.

For transcriptional activity, *Bacillus subtilis* strains (WT and MP2) were cultivated in LB or MM medium with the defined concentration of potassium as described above for growth rate measurements; the cell samples were collected in intervals before and after stress exposure, as indicated in the particular experiment (see *Results and Discussion*).

For 2DE analysis, *Bacillus subtilis* strains (WT and MP2) were cultivated in MM medium with 0.5 mM K^+^ as described above for growth rate measurements and harvested 60 min after stress exposure.

### *B. subtilis yxkO* knock-out mutant strain construction

For transfer of the insertional mutation of the mini-Tn*10* transposon to the *yxkO* gene from an asporogenic genetic background from a previously prepared mutant [15], the PBS1 lysate was prepared from the L-42 mutant, and *Bacillus subtilis* 168 was transduced. The transductants were selected on LB chloramphenicol plates (5 µg/ml). The classical transduction protocol was used [25]. Insertion of mini-Tn*10* into the *yxkO* gene in a particular clone was confirmed by PCR using primers designed for the *yxkO* gene and the transposon region, as well. The respective PCR product was confirmed by sequencing, and the mutant was named LD1.

For inactivation of the *yxkO* gene, an integrative vector, pMUTIN4 from *Bacillus* Genetic Stock Center (BGSC - ECE 139), was used. A fragment of the *yxkO* allele with a ribosome binding site was generated by PCR with the primers 5′- *GGAGGATCCATAACAGGACAATCAGCC*-3′ and *5* ′- *CCCGAATTCAGGAAAAGAAAGCAGAGGAG*-3′ (the *Eco*RI and *Bam*HI restriction sites for direct cloning into pMUTIN4 are underlined) and ligated into *Eco*RI-*Bam*HI-digested pMUTIN4. The ligation mixture was transformed into *E. coli* DH5α, and clones with pMP2 plasmid were selected on LB ampicillin plates (100 µg/ml). Presence of the *yxkO* allele fragment in the plasmid was confirmed by sequencing.

Circular pMP2 plasmid was then used for transformation into *Bacillus subtilis* SG64, and single-crossover recombinants were selected on LB plates supplemented with erythromycin (0.3 µg/ml). Correct insertion into the chromosome was confirmed in a selected isolate by PCR and sequencing and named MP2.

### *Bacillus subtilis* P*ctc* promoter probe mutant strain construction

The promoter region of the *ctc* gene [26] was generated by PCR with the primers 5′- *CATAGAATTCCCATTTTTCGAGGTTTAAATCCTT*-3′ and 5′- *TTTAGGATCCCGAGTAAAGTCCGTTCTTTCTT*-3′ (the *Eco*RI and *Bam*HI restriction sites for direct cloning into plasmid are underlined) and ligated to the same sites of the suicide pDG1661 plasmid, which possesses an insertion site to the *amy* locus (BGSC – ECE112).

The ligation mixture was transformed into *E. coli* DH5 α strain, yielding pJT2 plasmid by selecting on LB plates supplemented by ampicillin (100 µg/ml).

For *Bacillus subtilis* mutant strain (WT/P*ctc* and MP2/P*ctc*) construction, the plasmid was linearized by *Xho*I restriction enzyme (RE) and transformed into the *Bacillus subtilis* SG4 strain and the MP2 mutant strain prepared as above. Correct insertion into the *amy* locus and right orientation towards the reporter *lacZ* gene were confirmed by PCR and sequencing in double-crossover recombinants selected on LB plates supplemented with chloramphenicol (5 µg/ml).

### β - Galactosidase assay of *lac*Z transcriptional fusions

Samples were cultivated and collected at specified intervals. Cell samples were permeabilized with lysozyme, and galactosidase activities were measured at OD_420_ and expressed in Miller units (M.U.) according to the protocol from the *Bacillus* Genetic Stock Center (BGSC) Catalog of Integration Vectors (http://www.bgsc.org/_catalogs/Catpart4.pdf), page 15.

### 2DE sample preparation

For one experiment, WT and MP2 mutant were grown and stressed in parallel, and cells were cultivated as specified in the *Growth condition* section. Cells were harvested by centrifugation (4000×g, 4°C, 5 min), washed with 0.01 M Tris/KCl buffer pH 8.0, resuspended in 1 ml of the same buffer with protease inhibitors (Sigma), and disrupted by sonication. The cell debris was removed by centrifugation (15,000×g, 4°C, and 10 min), and protein concentration in the supernatant was estimated with the BCA Protein Assay Kit – Reducing Agent Compatible (Pierce). The cell lysates were then treated with the Bio-Rad Ready Prep 2-D Cleanup Kit and diluted in an appropriate volume of rehydration buffer up to 100 µg of total protein per sample. From each growth condition (WT, WT stressed, MP2, MP2 stressed), samples in technical triplicates were prepared.

### 2D polyacrylamide gel electrophoresis and protein visualization

Each sample prepared above was loaded onto an IPG strip pH 4–7 (Bio-Rad). Isoelectric focusing (IEF) was performed after passive overnight rehydration at room temperature using voltage that linearly increased to steady state [100 V for 2 h (slow), 300 V for 2 h (slow), 8000 V for 2 h slow, 8000 V for 7 h (rapid), and finished at 500 V] using the Bio-Rad Protean IEF system. After IEF, the strips were washed in equilibration solution (50 mM, Tris-HCl (pH 6.8), 6 M urea, 30% glycerol and 2% SDS) containing 0.02 M DTT for 10 min, followed by a second 10-min wash in equilibrium buffer containing 0.025 M iodoacetamide and bromophenol blue for gel staining.

The separation in the second dimension was carried out using precast gradient gels (Criterion Precast Gel (10.5%–14%) Bio-Rad). Gels were run on the Bio-Rad Criterion Dodeca Cell device at 5 V for 30 min and at 100 V for 2 hours at room temperature. Gels from one independent experiment in technical triplicates for each stress condition were run together (12 gels).

After staining with Colloidal Coomassie G-250 (Simply Blue Safestain, Invitrogen Life technologies, Paisley, UK), the gels were scanned with a GS-800 calibrated densitometer.

### Digitalization of gel images and data analysis

The 2DE image analysis was performed using PDQuest 8.0 software (Bio-Rad, Hercules, CA). For matching and quantification, raw images were smoothed to remove noise, background was subtracted, and a spot-by-spot visual validation of automated analysis was done from then on to increase the reliability of the matching. Only spots that exhibited similar intensity in each gel of the technical triplicates in particular growth condition and in both biological replicates were taken for further analysis. Data from PDQuest analysis are published in Table S1. Identified protein spots were manually cut out of the gels and analyzed by MS.

### MS analysis

The bands of interest were cut out of the gels and chopped into 1×1×1-mm pieces. The pieces were destained; to reduce and block cysteines, DTT and iodoacetamide were applied. The samples were trypsinized as described previously [27]. The dried-droplet method of sample preparation was employed, and spectra were acquired on a 4800 Plus MALDI TOF/TOF analyzer (AB Sciex). The data were analyzed using in-house running Mascot server 2.2.07 and matched against the current release of the NCBI protein sequence database: taxonomy: Bacillus (*Taxonomy ID:* 1386), 833049 sequences, and protein scores greater than 72 were significant (p<0.05). Cysteine carbamidomethylation, methionine oxidation, and N, Q deamination were set as fixed or variable modifications, respectively. One missed cleavage site was allowed. Precursor accuracy was set to 50 ppm, and the accuracy for MS/MS spectra was set to 0.25 Da. Detailed results are collected in Table 2.

**Table 2:** MALDI-TOF peptide mapping identification.

Protein	Protein Accession	MascoteScore	Best Protein Description
**Osmotic stress**			
Pgk	gi|16080446	104	phosphoglycerate kinase [Bacillus subtilis subsp. subtilis str. 168]
CitC	gi|16079965	885	isocitrate dehydrogenase [Bacillus subtilis subsp. subtilis str. 168]
Mdh	gi|16079964	105	malate dehydrogenase [Bacillus subtilis subsp. subtilis str. 168]
Mdh deg	gi|16079964	150	malate dehydrogenase [Bacillus subtilis subsp. subtilis str. 168]
AtpD iso1	gi|16080734	93	F0F1 ATP synthase subunit beta [Bacillus subtilis subsp. subtilis str. 168]
AtpD iso 2	gi|16080734	215	F0F1 ATP synthase subunit beta [Bacillus subtilis subsp. subtilis str. 168]
AtpD iso 3	gi|16080734	335	F0F1 ATP synthase subunit beta [Bacillus subtilis subsp. subtilis str. 168]
FrlB	gi|16080314	97	hypothetical protein BSU32610 [Bacillus subtilis subsp. subtilis str. 168]
Hag iso1	gi|16080589	83	flagellin [Bacillus subtilis subsp. subtilis str. 168]
Hag iso2	gi|16080589	78	flagellin [Bacillus subtilis subsp. subtilis str. 168]
Hag iso 3	gi|16080589	83	flagellin [Bacillus subtilis subsp. subtilis str. 168]
GroEL	gi|16077670	74	chaperonin GroEL [Bacillus subtilis subsp. subtilis str. 168]
GlnA	gi|16078809	123	glutamine synthetase [Bacillus subtilis subsp. subtilis str. 168]
**Ethanol stress**			
Pgk	gi|16080446	329	phosphoglycerate kinase [Bacillus subtilis subsp. subtilis str. 168]
CitC	gi|16079965	118	isocitrate dehydrogenase [Bacillus subtilis subsp. subtilis str. 168]
Mdh	gi|16079964	129	malate dehydrogenase [Bacillus subtilis subsp. subtilis str. 168]
Mdh deg	gi|16079964	209	malate dehydrogenase [Bacillus subtilis subsp. subtilis str. 168]
Atp iso1	gi|16080734	125	F0F1 ATP synthase subunit beta [Bacillus subtilis subsp. subtilis str. 168]
Atp iso 2	gi|16080734	155	F0F1 ATP synthase subunit beta [Bacillus subtilis subsp. subtilis str. 168
AtpD iso 3	gi|16080734	341	F0F1 ATP synthase subunit beta [Bacillus subtilis subsp. subtilis str. 168
FrlB	gi|16080314	119	fructoselysine-6-P-deglycase [Bacillus subtilis subsp. subtilis str. 168]
Hag iso1	gi|16080589	157	flagellin [Bacillus subtilis subsp. subtilis str. 168]
Hag iso2	gi|16080589	175	flagellin [Bacillus subtilis subsp. subtilis str. 168]
Hag iso 3	gi|16080589	89	flagellin [Bacillus subtilis subsp. subtilis str. 168]
GroEL	gi|16077670	140	chaperonin GroEL [Bacillus subtilis subsp. subtilis str. 168]
Ilvc iso1	gi|16079881	194	ketol-acid reductoisomerase [Bacillus subtilis subsp. subtilis str. 168]
Ilvc iso2	gi|16079881	183	ketol-acid reductoisomerase [Bacillus subtilis subsp. subtilis str. 168]
Asd	gi|16078738	81	aspartate-semialdehyde dehydrogenase [Bacillus subtilis subsp. subtilis str. 168]
Ald	gi|16080244	79	L-alanine dehydrogenase [Bacillus subtilis subsp. subtilis str. 168]

Detailed data from MALDI-TOF peptide mapping identification of proteins, that differed in levels when exposed to osmotic and ethanol stress.

## Results and Discussion

### Confirmation of *yxkO* knock-out mutant phenotype

**Figure 1:**
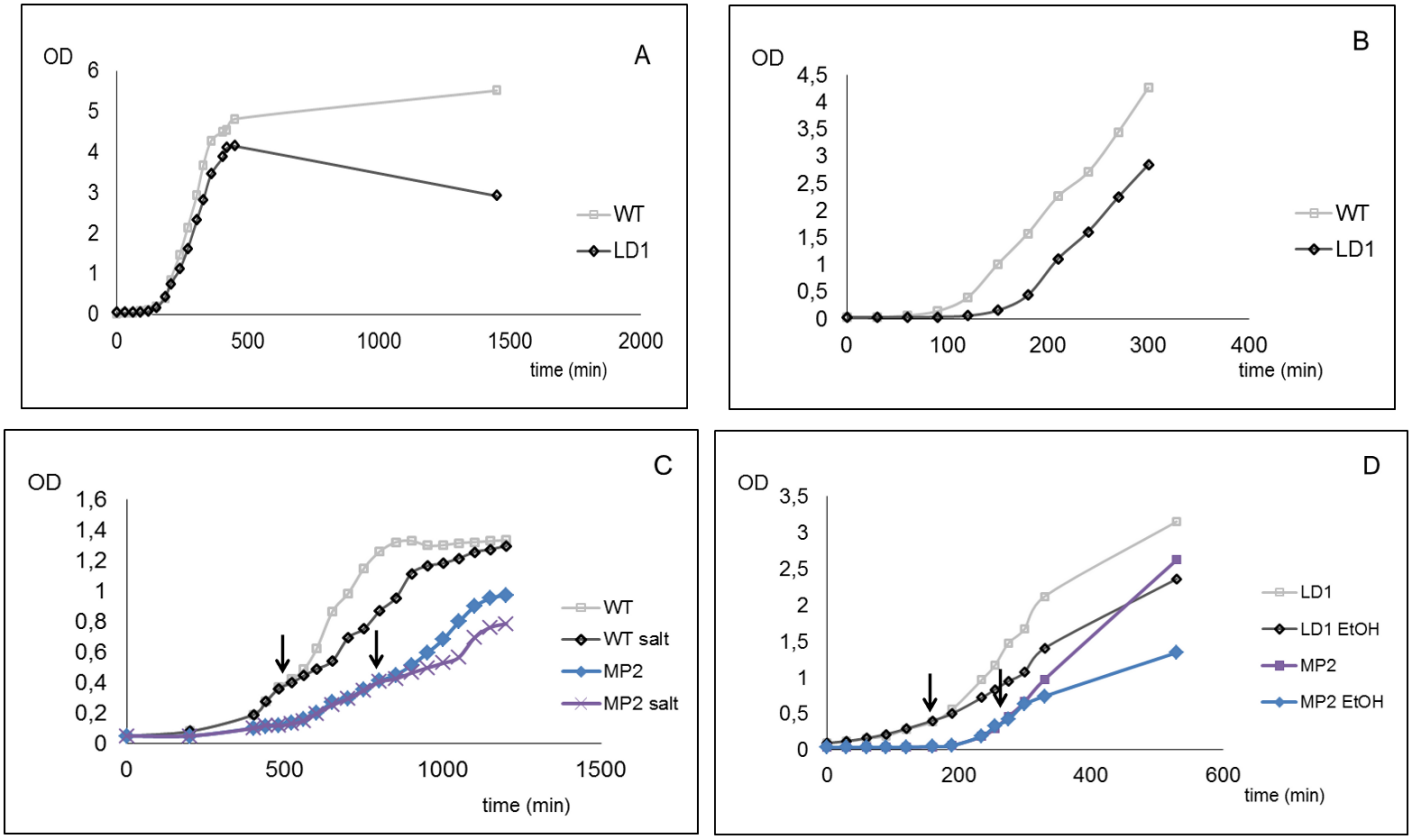
Growth characterization of WT, LD1, and MP2 mutants of *Bacillus subtilis* in long-term cultivation and in response to stress. For long-term growth measurements, the cells were grown in LB medium (A). Effect of MM medium and K^+^ limitation (0.5 mM K^+^) to growth rate of mutant (LD1) (B). Osmotic stress performed only for MP2 mutant in MM medium and K^+^ limitation is demonstrated (C). Effect of ethanol stress was measured in MM medium and K+ limitation for both mutants (LD1, MP2) (D). (WT - *Bacillus subtilis* 168 or SG4, LD1 and MP2– *yxkO* knock-out mutants, for details see Table 1). Exposure of stress is marked by arrows. Measurements were done with cells synchronized in exponential growth, and stress conditions were set up as is described in *Material and Methods*. The typical growth rate curve is shown from measurements made in triplicate for each condition.

Firstly, to verify the *yxkO* gene phenotype previously described in a sporulation-proficient strain (*spo0F221*) [15], we transduced the cassette of mini-Tn*10* from the L-42 mutant using PBS1 bacteriophage to *Bacillus subtilis* 168, and in parallel, we constructed an insertional mutant using the pMUTIN4 plasmid, as well (see *Materials and Methods*). Both mutants revealed the previously described phenotype, an increase in generation time from 55 min to 115 min and osmosensitivity in low K^+^ conditions (15) but also lower viability, even under stress conditions, as we demonstrated in the long-term growth measurements (Figure 1A).

By doing so, we confirmed that the previously described phenotypic changes in mutant L-42 corresponded to the inactivation of the *yxkO* gene and not to the *spo0F* mutation. Both types of the mutant revealed prolonged lag phase (Figure 1B), and lower viability when exposed to ethanol and salt stress when cultivated in media with a potassium concentration lower than 1 mM (0.5 mM) (Figure 1C, D). Both types of mutants revealed a similar course of the growth curves.

### Characterization of *yxkO* gene promoter transcriptional activity under stress conditions

As the next routine step in characterization of the *yxkO* mutant, we tested activity of the P*yxkO* promoter when exposed to several stress conditions. For this experiment, we used the previously prepared MP2 mutant, as the plasmid pMUTIN4 was constructed to be used for gene knock-out and as a promoter probe with the *lac*Z reporter, as well, with the benefit of complementation of the WT allele. Surprisingly, no significant increase of transcription was observed under any cultivation condition and induction of the WT allele with IPTG, respectively (low, high concentration of K^+^, salt stress), except for ethanol stress, when a slight increase of transcription was detected after the stress exposure (data not shown). The same result was observed previously, when transcriptional activity was tested from solely promoter cloned to pDG1661promoter probe vector (unpublished data). These data indicate that the protein is expressed only at low levels or undergoes more complex regulation of transcription.

### Testing of YxkO’s impact on activation of general stress response

**Figure 2:**
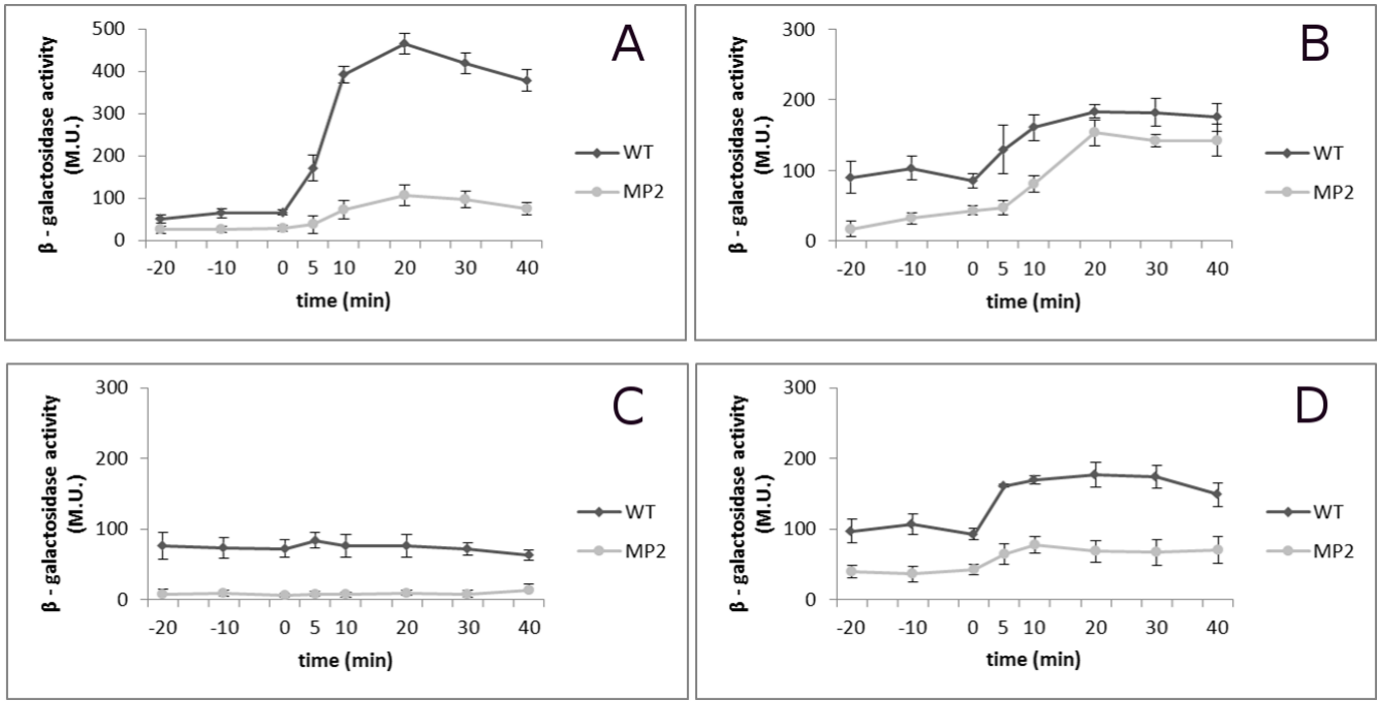
Transcription level of P*ctc* on genetic background of WT and MP2 mutant. P*ctc* activation measurements of WT and MP2 were performed under ethanol stress in LB medium (A), osmotic stress in MM medium with 10 mM K^+^ concentration (B), osmotic stress in MM medium with 0.5 mM K^+^ concentration (C), shift from MM medium with 10 mM K^+^ concentration to MM medium with 0.5 mM K^+^ concentration (D). Time 0 indicates application of stress. Details of transcription activity measurements, growth, and stress conditions are described in *Material and Methods.*

When direct estimation of the promoter activity of the P*yxkO* promoter had failed, we tested the impact of the YxkO on the induction of SigB-dependent genes. For this purpose, we generally used the well-characterized SigB-dependent P*ctc* promoter, which is transcribed under the most studied stress conditions, and the P*ctc* promoter fused to *lac*Z (P*ctc*-*lac*Z) beneficially monitors SigB activity in the WT promoter context, as was shown previously [11]. The promoter-probe strains in both genetic backgrounds were prepared as described in the *Materials and Methods* section, and the promoter activity of P*ctc* was monitored. The tested conditions were ethanol stress in complex media and salt stress when cultivated in high (10 mM) and low (0.5 mM) concentrations of K^+^, respectively. Results are shown in Figure 2.

When exposed to ethanol stress, the promoter activity of P*ctc* in the MP2 mutant was markedly lower (Figure 2A). In the high K^+^ concentration condition assay, the activity of P*ctc* increased in WT and the MP2 mutant after the salt stress, as well, albeit at a lower level in the mutant (Figure 2B). When cells were cultivated in medium with low K^+^ concentration, the transcription from P*ctc* revealed a completely different course. The transcription level was high in WT, even before salt stress, and remained low in the mutant, even after the stress exposure (Figure 2C).

Therefore, we monitored the promoter activity of P*ctc* when cells were cultivated first in the medium with high K^+^ concentration and then shifted to medium with low K^+^ concentration. In both WT and the mutant strain, an increase in the P*ctc* activity occurred after the K^+^ concentration shift but in the mutant the promoter activity was systematically lower at all-time points (Figure 2D). As mentioned above, a 1 mM concentration of K^+^ limits the potassium KtrAB transport system of *Bacillus subtilis* 168, which is predicted to be involved in osmoadaptation [28].

These results indicated that in *Bacillus subtilis*168, a K^+^ concentration in the medium below 1 mM is a stress condition and causes the general stress response.

The different level of P*ctc* transcription in the presented experiments is in agreement with the recent observation of Young et al. that the extent of the stress causes a differential effect on the adaptation response in subpopulations of *Bacillus subtilis* [14]. It is obvious that potassium deficiency coupled with osmotic stress causes a distinct adaptive response than the osmotic stress itself. As the product of the *yxkO* gene is assumed to be NADH hydrate dehydratase, it is tempting to speculate about the role of energy imbalance in the activation of the SigB regulon and SigB itself.

### Comparative 2DE analysis of YxkO impact on an adaptive response to stress

We compared protein levels of cytoplasmic proteins in the pI range of 4–7 in exponentially growing cells of WT and the yxkO knock-out mutant (MP2) of *Bacillus subtilis* in medium with limited potassium and exposed to osmotic and ethanol stress, respectively. Cells were grown and analyzed by comparative 2DE as described in *Materials and Methods*.

**Figure 3:**
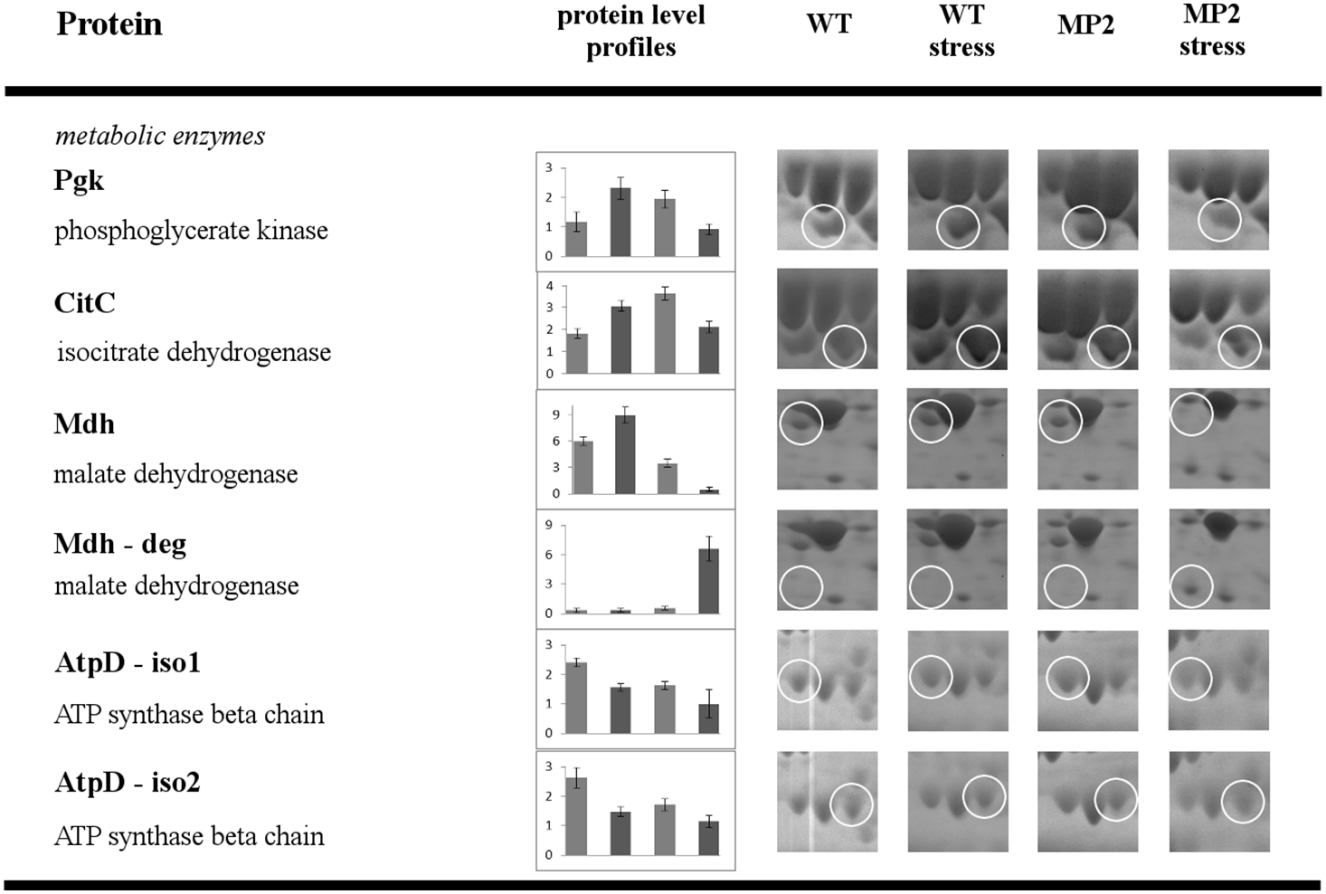
Comparative 2DE analysis of WT versus MP2 mutant exposed to osmotic and ethanol stress – metabolic enzymes. Proteins with protein level profiles that are similar for both stresses. For experimental conditions and data evaluation, see *Material and Methods*. Separate columns of the bar charts show the protein level of respective proteins, as calculated from the quantification of the spot volume by PDQuest 8.0 software; y-axes are scaled in intensity for each particular protein. Bars represent each strains and conditions, and there are in the same order as the protein level profiles are presented.

**Figure 4:**
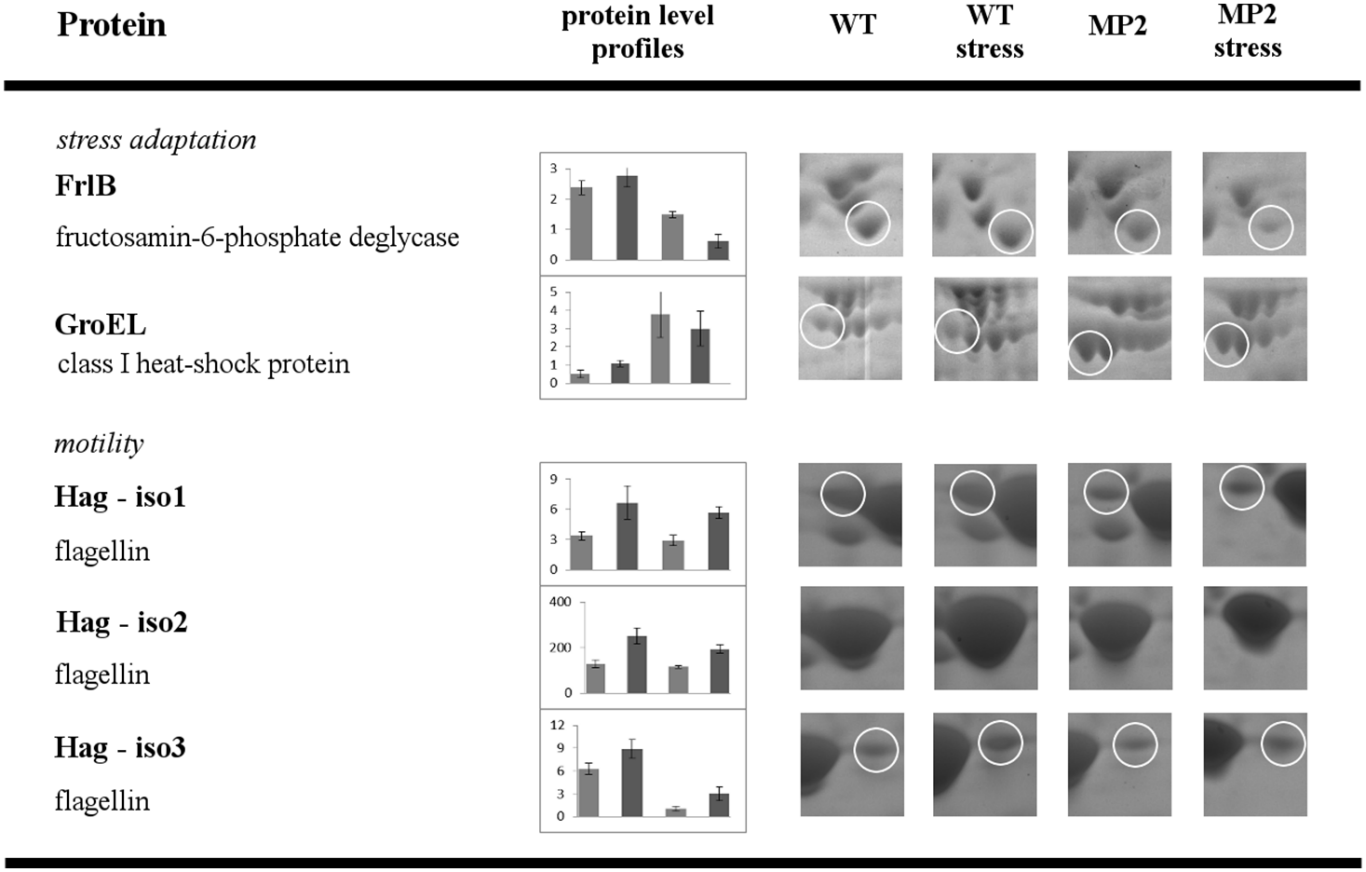
Comparative 2DE analysis of WT versus MP2 mutant exposed to osmotic and ethanol stress – stress adaptation and motility. Proteins with protein level profiles that are similar for both stresses. For experimental conditions and data evaluation, see *Material and Methods*. The picture description is same as for Figure 3.

**Figure 5:**
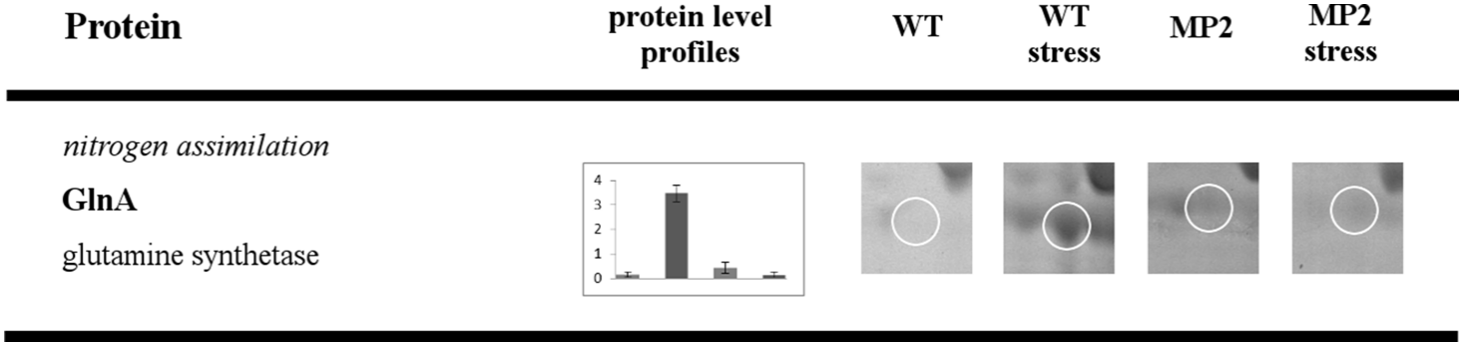
Comparative 2DE analysis of WT versus MP2 mutant exposed to osmotic stress. Proteins with protein level profiles that are unique for osmotic stress. For experimental conditions and data evaluation, see *Material and Methods*. The picture description is same as for Figure 3.

**Figure 6:**
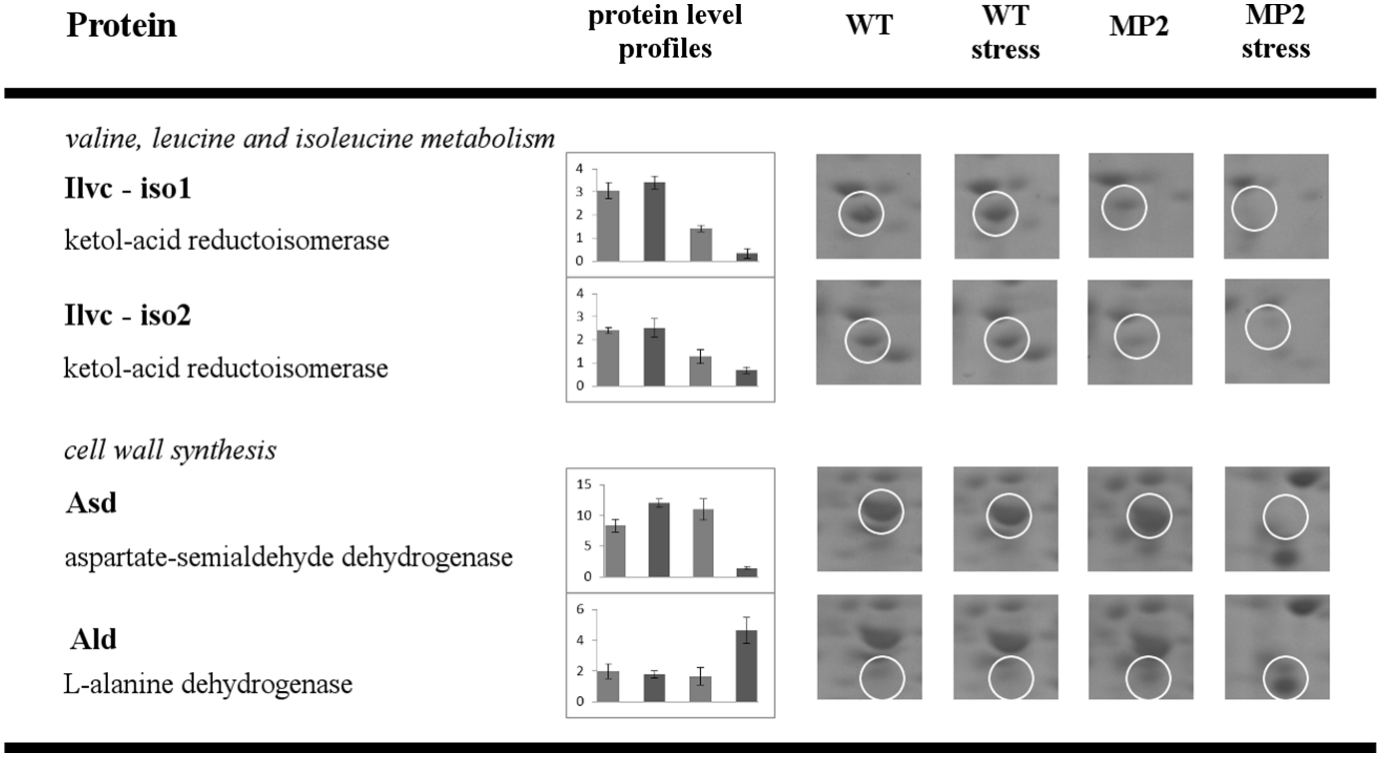
Comparative 2DE analysis of WT versus MP2 mutant exposed to ethanol stress. Proteins with protein level profiles that are unique for ethanol stress. For experimental conditions and data evaluation, see *Material and Methods*. The picture description is same as for Figure 3.

The results, summarized in Figures 3–6, show that disruption of the *yxkO* gene caused changes in the level of seven proteins belonging to various metabolic pathways upon both stresses (Figure 3 and Figure 4), and we detected only one protein with a different intensity solely under osmotic stress (Figure 5), three enzymes revealed an altered pattern merely under ethanol stress (Figure 6). Figure S1 illustrates the pattern of the 2DE spot distribution.

### Proteins with levels that differ under both stresses

#### Metabolic enzymes

It is described elsewhere that after osmotic stress, a prompt decrease in expression of most of the glycolytic and citrate cycle enzymes occurs and, in intervals to 60 min, increases to initial levels after resumption of growth arises [5]. Our results are in agreement with this, as we determined the different levels of phosphoglycerate kinase, an enzyme representing the glycolytic pathway, and as we detected changes in the protein level of isocitrate dehydrogenase and malate dehydrogenase from citrate cycle enzymes, as well. In the mutant, 60 minutes after stress exposure, the protein level remained low in the case of all three mentioned proteins, and we detected degradation products of malate dehydrogenase (Mdh-deg, MP2 stress sample) in the case of EtOH stress (Figure 3). There is no study in the literature about the mechanism that is involved in the cessation and recovery of expression of these three vegetative proteins after osmotic stress, but the role of the *yxkO* gene is evident from our experiment.

This effect highlights the recently recognized biochemical activity of YxkO protein as an enzyme that regenerates the hydrated form of NAD(P)H emerging in cells during stress [17]. The absence of dehydration of NADHX and the regeneration of activities of both isocitrate dehydrogenase and malate dehydrogenase may result in energetic imbalance and, subsequently, growth retardation and reduced stress adaptation in the MP2 mutant, as we documented by extension of the lag phase of stressed cultures of mutant cells.

We also detected changes in the protein level of the ATPase subunit, similar to Höper’s results [5], and we determined three isoforms of the beta subunit, AtpD. The protein levels of two isoforms (iso1, iso2) manifested the same pattern as in the case of metabolic enzymes, and recovery in the mutant did not occur, either. After stress, there was a significant decrease in the protein level of both isoforms in the mutant (Figure 3).

#### Stress adaptation

Another protein that was determined to be differentially accumulated under both stresses was fructosamine-6-phosphate deglycase (FrlB, formerly YurP); the protein level increased after the stress in the WT and this increase was absent in the mutant, regardless of stress exposure (Figure 4). This gene was also detected to be upregulated under most stress conditions at the transcription level [4] and was shown to be under direct regulation of the CodY general regulator [29]. This enzyme catalyzes the cleavage of fructosamine-6-phosphate to glucose-6-phosphate and the corresponding amines in *Escherichia coli* and *Bacillus subtilis* and is involved in enzymatic deglycation of Amadori products [30]. Intracellular glycation has been described to occur in *Escherichia coli* [31], and it is very likely to occur in *Bacillus subtilis*, as well. DNA-binding activity of CodY is regulated by the intracellular level of GTP [32], and it was evidenced that during nutrient starvation, the ratio of ATP/GTP is changed [33]. In environmental stress adaptation, this phenomenon has not yet been studied, but it could be supposed that inactivation of NADH-synthesizing enzymes gives rise to an energy imbalance, changing the ATP/GTP ratio, as well.

Changes in the protein value caused by *yxkO* disruption were also recorded for GroEL (Figure 4). In the mutant, the increase of protein level occurred in non-stressed conditions, as well when compared to the WT. This can be explained by extension of the lag phase and the decline of the renewing of isocitrate dehydrogenase levels in the mutant according to WT after stress exposure, which denotes to failure of stress adaptation and triggers increased levels of GroEL as a result of the devastating effects of both stresses on cellular proteins.

#### Motility

Another protein that differed in protein level in WT versus MP2 and the changes of which corresponded to the mutant phenotype that the *yxkO* gene disruption affected is flagellin (Figure 4). When the cells were cultivated under a limited concentration of potassium, there was massive expression of flagellin protein. This corresponds to the rapid movement and substantial flagellation of cells observed in light and electron microscopy, respectively. In native microscopic preparates, we also observed different motility of WT and mutant cells before and after being subjected to salt stress. It has been described that after osmotic shock, a decrease in the transcription of genes coding for motility apparatus components occurs [1]. In agreement with Steiĺs results, we have observed that after the shock, the WT cells ceased to move in the adaptation phase, whereas the mutant cells did not show any significant changes in their motility, regardless of the stress (data not shown).

On our gels, we detected three isoforms of flagellin, with the evidence that the formation of quantitatively less represented isoforms (iso1 and iso3) was affected by the disruption of the *yxkO* gene. A drop in other proteins of the flagellar apparatus, such as basal body components, was not detected. This could indicate that the product of the *yxkO* gene does not influence the intracellular amount of flagellin protein but causes its modification, which allows or prevents the transport of monomers out of the cell and its degradation in the cytoplasm, respectively. The origins and nature of these isoforms of flagellin have not been described in the literature to date and require further work to be elucidated. We can speculate that non-enzymatic glycation (see FrlB above) is involved.

### Protein with levels that differ solely under osmotic stress

#### Nitrogen assimilation

Glutamine synthetase, the key enzyme of nitrogen assimilation, is a protein, the level of which was affected by mutation in the *yxkO* gene only after osmotic stress (Figure 5). We determined a massive increase in the protein amount of this enzyme in WT after the salt stress, while in the MP2 mutant, the protein stayed at a low level, even after the stress. Our results differ from those of Höper [5], which can be explained by the fact that we used a mineral medium with ammonium sulphate as the sole source of nitrogen and a limited concentration of potassium, conditions under which nitrogen metabolism are also significantly influenced by CodY and ATP assistance, as was recently reported by Gunka [34].

### Proteins with a different level under ethanol stress

A comprehensive proteomic study of *Bacillus subtilis* under ethanol stress with which we could compare our results has not yet been published. After the ethanol stress, we observed changes in a similar set of proteins as in the salt stress experiment, with a comparable intensity profile (see above). However, we identified proteins, the accumulation of which was influenced only after the ethanol stress (Figure 6). Changes were detected in the protein amount of three enzymes: ketol-acid reductoisomerase (IlvC), aspartate-semialdehyde dehydrogenase (Asd) and amino acid L-alanine dehydrogenase (Ald).

#### Valin, leucine, and isoleucine metabolism

The first enzyme, ketol-acid reductoisomerase (IlvC), is known to be involved in the biosynthesis of valine, leucine, and isoleucine, and its encoding gene is a part of the *ilv-leu* operon [35]. We identified that the cellular level of IlvC decreased in the mutant after the stress, while in WT, it remained unchanged. Valine, leucine, and isoleucine are the precursors for the biosynthesis of iso- and anteiso-branched fatty acids, which represent the major fatty acid species of membrane lipids in *Bacillus* sp. The increase in their expression was proposed as a long-term adaptation mechanism to cold and ethanol stresses [36]. It is worth mentioning that as already reported by Molle [37], the *ilv-leu* operon was shown to be controlled by the global transcriptional regulator CodY.

#### Cell wall synthesis

The second enzyme, aspartate-semialdehyde dehydrogenase (Asd), the expression of which was lower after the stress in the mutant, is involved in the metabolism of alanine, aspartate, and glutamate. The third enzyme, amino acid L-alanine dehydrogenase (Ald), catalyzes deamination of alanine to pyruvate. Its level in the mutant strain was markedly higher after the stress, while in WT, it was not affected. Both are the precursors for the synthesis of diaminopimelic acid, which is a constituent of peptidoglycan. It has been reported that ethanol stress increases the expression of proteins involved in cell wall synthesis [38], and phosphorylation of aspartate-semialdehyde dehydrogenase by PtkA kinase was described [39]. An imbalance in expression of this protein may cause a defect in cell wall synthesis in the mutant strain and consequently decrease its adaptability to ethanol stress.

## Conclusions

In this study, we performed the first characterization of a knock-out mutant in the *yxkO* gene, which biochemical activity was classified *in* *vitro* as an NAD(P)H hydrate dehydratase, and provided evidence that it is involved in stress adaptation (to osmotic and ethanol stress and under potassium limitation).

Our results correspond to the observed phenotype of the mutant and show that stress adaptation reactions in the mutant are merely suppressed or not activated, as reflected by the observed decline in the growth and extension of the lag phase, inhibition of recovery of NADH-dependent proteins during the lag phase after stress exposure (Idh, Mdh, etc.), energy imbalance and repression of CodY-regulated proteins (FrlB, IlvC), and inhibition of activation of a general stress protein (Ctc) in the mutant. We have also shown that potassium limitation is a stress condition for *Bacillus subtilis* that activates the general stress response. This reflects the complexity and interconnection of stress adaptation processes and is evidence that either adaptation mechanism is important in stress resistance. Thus metabolite repair enzymes play a significant role in the survival of stressed cells.

## Supporting Information

Figure S1 **The 2-DE image of cytoplasmic proteins from WT Bacillus subtilis 168 cultivated in the ethanol stress condition, illustrating the pattern of the 2DE spot distribution.** The patterns of the 2DE spot distributions in the studied culture conditions of WT and MP2 mutant were similar. Proteins that exhibited changes in protein abundances and were identified by MS MALDI-TOF are pointed out. Details of identified proteins are described in *Results and Discussion*, Figures 3–6, and Table 2.
(TIF)

Table S1 **Supplemental tables with data of spots quantification.**
(XLSX)
