# Supplementary figures and images for: NAD(P)H-Hydrate Dehydratase- A Metabolic Repair Enzyme and Its Role in Bacillus subtilis Stress Adaptation
Source: PLoS One. 2014 Nov 13;9(11):e112590. doi: 10.1371/journal.pone.0112590 (PMC4231035; doi:10.1371/journal.pone.0112590)

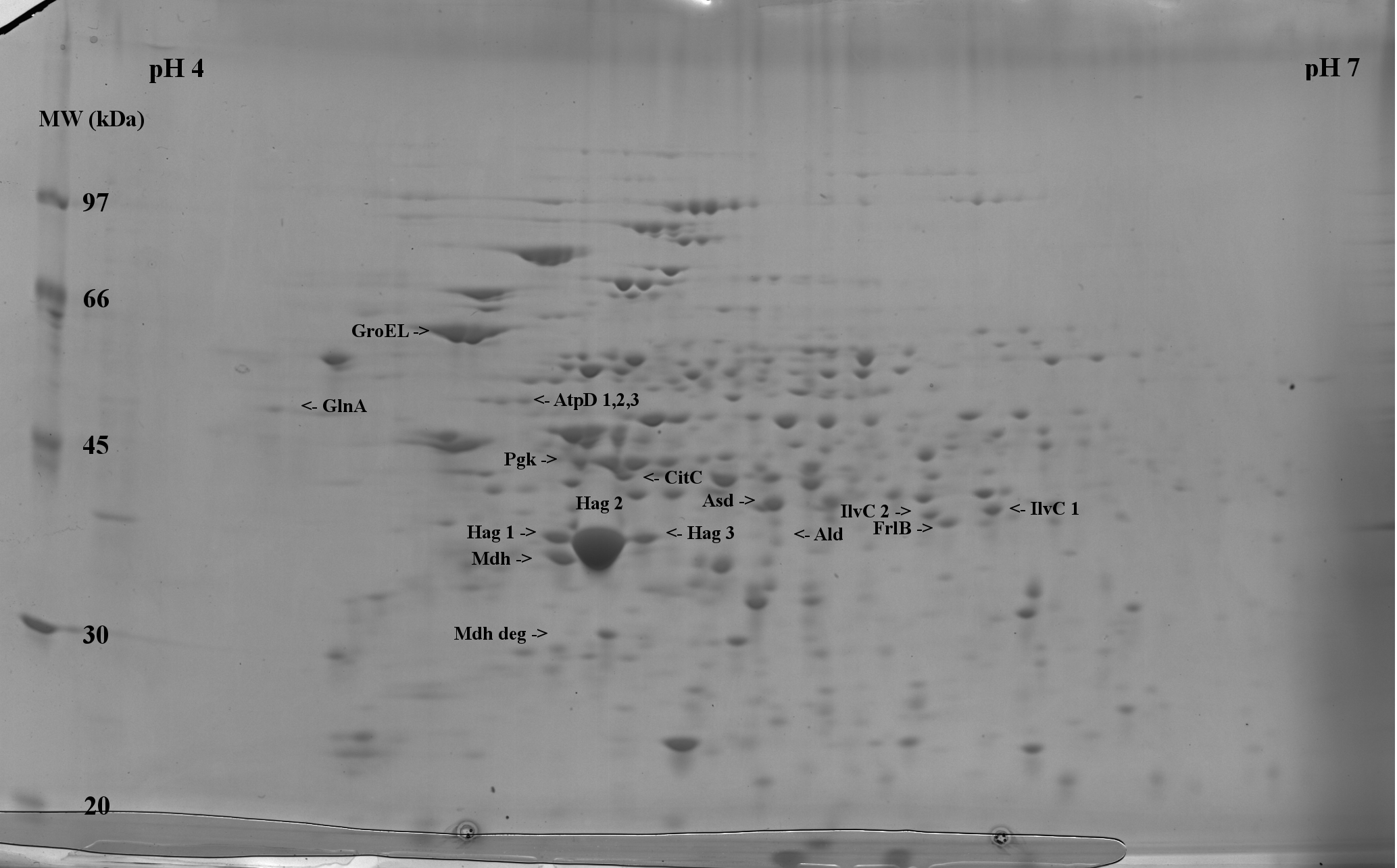

Supplement: Figure S1 — The 2-DE image of cytoplasmic proteins from WT Bacillus subtilis 168 cultivated in the ethanol stress condition, illustrating the pattern of the 2DE spot distribution. The patterns of the 2DE spot distributions in the studied culture conditions of WT and MP2 mutant were similar. Proteins that exhibited changes in protein abundances and were identified by MS MALDI-TOF are pointed out. Details of identified proteins are described in Results and Discussion, Figures 3–6, and Table 2. (TIF) [file pone.0112590.s001.tif]
